# Bis[μ-1,2-bis­(1,2,4-triazol-4-yl)ethane]bis­[diiodidozinc(II)]

**DOI:** 10.1107/S1600536810014121

**Published:** 2010-04-24

**Authors:** Yunfei Feng, Na Liang, Baolong Li, Haiyan Li

**Affiliations:** a College of Chemistry and Chemical Engineering and Material Science, The Key Laboratory of Organic Synthesis of Jiangsu Province, Suzhou University, Suzhou 215123, People’s Republic of China

## Abstract

In the title dinuclear complex, [Zn_2_I_4_(C_6_H_8_N_6_)_2_], two Zn^II ^atoms are bridged by two 1,2-bis­(1,2,4-triazol-4-yl)ethane (btre) ligands, forming a centrosymmetric metallacycle. The coordination geometry of the Zn^II^ ion is distorted tetra­hedral with the coordination sphere formed by two N atoms from the triazole rings of two symmetry-related btre ligands and two iodide ligands.

## Related literature

For the isostructural zinc complexes [Zn_2_(btre)_2_ *X*_4_], where *X *= Cl, Br, see: Habit *et al.* (2009 ▶). For other triazole coordin­ation compounds, see: Haasnoot (2000 ▶); Li *et al.* (2003 ▶); Zhang *et al.* (2007 ▶); Zhu *et al.* (2004 ▶).
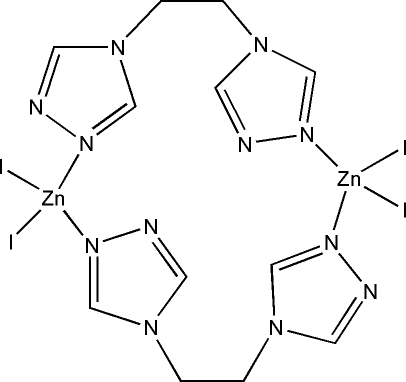


## Experimental

### 

#### Crystal data

[Zn_2_I_4_(C_6_H_8_N_6_)_2_]
*M* *_r_* = 966.71
Monoclinic, 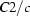

*a* = 20.241 (5) Å
*b* = 7.3847 (14) Å
*c* = 17.348 (4) Å
β = 97.375 (5)°
*V* = 2571.6 (9) Å^3^
*Z* = 4
Mo *K*α radiation
μ = 6.69 mm^−1^
*T* = 293 K
0.59 × 0.21 × 0.20 mm

#### Data collection

Rigaku Mercury CCD diffractometer
Absorption correction: multi-scan (*REQAB*; Jacobson, 1998 ▶) *T*_min_ = 0.110, *T*_max_ = 0.348
11703 measured reflections
2339 independent reflections
2063 reflections with *I* > 2σ(*I*)
*R*_int_ = 0.040

#### Refinement

*R*[*F* ^2^ > 2σ(*F* ^2^)] = 0.039
*wR*(*F* ^2^) = 0.106
*S* = 1.07
2339 reflections
136 parameters
H-atom parameters constrained
Δρ_max_ = 0.69 e Å^−3^
Δρ_min_ = −1.31 e Å^−3^

### 

Data collection: *CrystalClear* (Rigaku, 2000 ▶); cell refinement: *CrystalClear*; data reduction: *CrystalClear*; program(s) used to solve structure: *SHELXS97* (Sheldrick, 2008 ▶); program(s) used to refine structure: *SHELXL97* (Sheldrick, 2008 ▶); molecular graphics: *SHELXTL* (Sheldrick, 2008 ▶); software used to prepare material for publication: *SHELXTL*.

## Supplementary Material

Crystal structure: contains datablocks global, I. DOI: 10.1107/S1600536810014121/gk2262sup1.cif

Structure factors: contains datablocks I. DOI: 10.1107/S1600536810014121/gk2262Isup2.hkl

Additional supplementary materials:  crystallographic information; 3D view; checkCIF report

## Figures and Tables

**Table:** 

Zn1—N1	2.017 (5)
Zn1—N4^i^	2.019 (5)
Zn1—I1	2.5479 (8)
Zn1—I2	2.5523 (9)

**Table:** 

N1—Zn1—N4^i^	103.68 (19)
N1—Zn1—I1	108.78 (14)
N4^i^—Zn1—I1	112.08 (14)
N1—Zn1—I2	113.03 (14)
N4^i^—Zn1—I2	107.90 (14)
I1—Zn1—I2	111.19 (3)

Symmetry code: (i) 
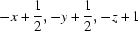
.

**Fig. 1.**
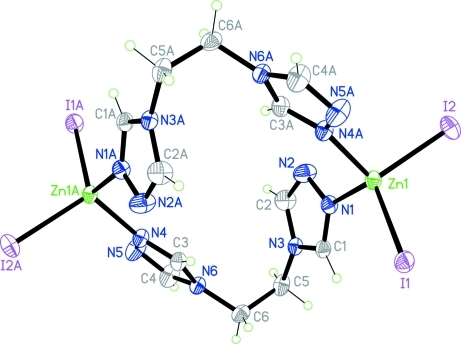
A dimeric structure of the title compound. Displacement ellipsoids are drawn at the 30% probability level (symmetry code for atoms A: -x+1/2, -y+1/2, -z+1).

**Fig. 2.**
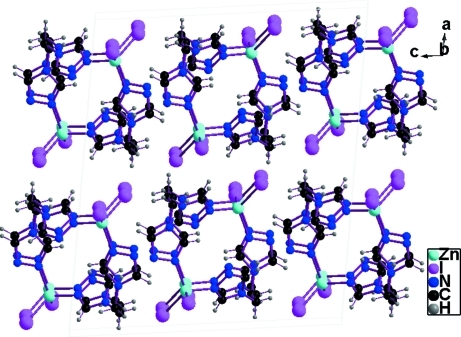
Crystal packing of the title compound viewed along the [010] direction.

## supplementary crystallographic information

### Comment

A large number of mononuclear, oligonuclear and polynuclear transition metal complexes of 1,2,4-triazole derivatives have been synthesized and characterized because of their magnetic properties and novel topologies (Haasnoot, 2000).

In our previous work, we synthesized several zinc^II^ complexes with 1,2-bis(1,2,4-triazol-1-yl)ethane (bte; Li *et al.*, 2003; Zhang *et al.*, 2007; Zhu *et al.*, 2004). 1,2-Bis(1,2,4-triazol-4-yl)ethane (btre) is an isomer of 1,2-bis(1,2,4-triazol-1-yl)ethane. In the present work, we report here the preparation and crystal structure of a dimeric zinc^II^ complex, namely, [Zn(btre)I_2_]_2_ (I).

The crystal structure of (I) is built up from a neutral dimeric metallacycle. The dimer is centrosymmetric. As shown in Fig.1, in each dimer, two zinc^II^ centres are connected by two btre lignads resulting in a discrete Zn_2_(btre)_2_ 18-membered binuclear metallacycle.

Each zinc^II^ centre is four-coordinated by two N atoms of btre ligands and two I lignads (Table 1), forming a distorted tetrahedral geometry. Each btre exhibits a gauche conformation in (I). The N3—C5—C6—N6 torsion angle is 63.8 (7)°. The dihedral angle between the two triazole rings is 45.6 (2)°. The Zn···Zn separation via the bridging btre ligand is 7.755 (2) Å in (I), compared with the corresponding values of 7.8750 (2) Å in [Zn(btre)Cl_2_]_2_ and 7.7980 (5) Å in [Zn(btre)Br_2_]_2_ (Habit *et al.*, 2009).

### Experimental

10 ml of aqueous solution of ZnI_2_ (1 mmol) was added to a tube, and 10 ml of MeOH solution of 1,2-bis(1,2,4-triazol-4-yl)ethane (btre) (1.0 mmol) was carefully added above the aqueous solution. Colourless crystals were obtained after about two weeks. Anal. Calcd. for C_12_H_16_I_4_N_12_Zn_2_: C, 14.91; H, 1.67; N, 17.39%. Found: C, 14.82; H, 1.56; N, 17.31%.

### Refinement

H atom were placed in idealized positions and refined as riding, with C—H distances of 0.93 (triazole) and 0.97Å (ethane), and with U_iso_(H) = 1.2U_eq_(C).

### Crystal data

**Table:**

[Zn_2_I_4_(C_6_H_8_N_6_)_2_]	*F*(000) = 1776
*M**_r_* = 966.71	*D*_x_ = 2.497 Mg m^−^^3^
Monoclinic, *C*2/*c*	Mo *K*α radiation, λ = 0.71070 Å
Hall symbol: -c 2yc	Cell parameters from 4578 reflections
*a* = 20.241 (5) Å	θ = 3.1–25.4°
*b* = 7.3847 (14) Å	µ = 6.69 mm^−^^1^
*c* = 17.348 (4) Å	*T* = 293 K
β = 97.375 (5)°	Block, yellow
*V* = 2571.6 (9) Å^3^	0.59 × 0.21 × 0.20 mm
*Z* = 4	

### Data collection

**Table:**

Rigaku Mercury CCD diffractometer	2339 independent reflections
Radiation source: fine-focus sealed tube	2063 reflections with *I* > 2σ(*I*)
graphite	*R*_int_ = 0.040
ω scans	θ_max_ = 25.3°, θ_min_ = 3.1°
Absorption correction: multi-scan (REQAB; Jacobson, 1998)	*h* = −23→24
*T*_min_ = 0.110, *T*_max_ = 0.348	*k* = −8→8
11703 measured reflections	*l* = −20→20

### Refinement

**Table:**

Refinement on *F*^2^	Primary atom site location: structure-invariant direct methods
Least-squares matrix: full	Secondary atom site location: difference Fourier map
*R*[*F*^2^ > 2σ(*F*^2^)] = 0.039	Hydrogen site location: inferred from neighbouring sites
*wR*(*F*^2^) = 0.106	H-atom parameters constrained
*S* = 1.07	*w* = 1/[σ^2^(*F*_o_^2^) + (0.0585*P*)^2^ + 4.1632*P*] where *P* = (*F*_o_^2^ + 2*F*_c_^2^)/3
2339 reflections	(Δ/σ)_max_ = 0.001
136 parameters	Δρ_max_ = 0.69 e Å^−^^3^
0 restraints	Δρ_min_ = −1.31 e Å^−^^3^

### Special details

**Table:**

Geometry. All esds (except the esd in the dihedral angle between two l.s. planes) are estimated using the full covariance matrix. The cell esds are taken into account individually in the estimation of esds in distances, angles and torsion angles; correlations between esds in cell parameters are only used when they are defined by crystal symmetry. An approximate (isotropic) treatment of cell esds is used for estimating esds involving l.s. planes.
Refinement. Refinement of *F*^2^ against ALL reflections. The weighted *R*-factor wR and goodness of fit *S* are based on *F*^2^, conventional *R*-factors *R* are based on *F*, with *F* set to zero for negative *F*^2^. The threshold expression of *F*^2^ > σ(*F*^2^) is used only for calculating *R*-factors(gt) etc. and is not relevant to the choice of reflections for refinement. *R*-factors based on *F*^2^ are statistically about twice as large as those based on *F*, and *R*- factors based on ALL data will be even larger.

### Fractional atomic coordinates and isotropic or equivalent isotropic displacement parameters (Å^2^)

**Table:**

	*x*	*y*	*z*	*U*_iso_*/*U*_eq_	
Zn1	0.13008 (3)	−0.08434 (9)	0.58428 (4)	0.0339 (2)	
I1	0.05228 (2)	0.08983 (6)	0.66190 (3)	0.04764 (18)	
I2	0.09753 (2)	−0.41797 (6)	0.57188 (3)	0.05286 (19)	
N1	0.1311 (2)	0.0385 (7)	0.4806 (3)	0.0372 (11)	
N2	0.1752 (3)	−0.0096 (9)	0.4306 (3)	0.0571 (15)	
N3	0.1084 (2)	0.2012 (7)	0.3770 (2)	0.0340 (10)	
N4	0.2741 (2)	0.5707 (6)	0.3664 (3)	0.0375 (11)	
N5	0.2601 (3)	0.6027 (9)	0.2874 (3)	0.0580 (17)	
N6	0.1672 (2)	0.5550 (7)	0.3363 (3)	0.0365 (11)	
C1	0.0918 (3)	0.1627 (8)	0.4480 (3)	0.0361 (13)	
H1A	0.0573	0.2171	0.4702	0.043*	
C2	0.1604 (4)	0.0924 (10)	0.3697 (4)	0.0543 (19)	
H2A	0.1831	0.0903	0.3264	0.065*	
C3	0.2178 (3)	0.5456 (8)	0.3934 (3)	0.0389 (14)	
H3A	0.2135	0.5241	0.4453	0.047*	
C4	0.1964 (4)	0.5905 (10)	0.2722 (4)	0.057 (2)	
H4A	0.1732	0.6044	0.2227	0.069*	
C5	0.0761 (3)	0.3322 (10)	0.3219 (3)	0.0466 (16)	
H5A	0.0282	0.3217	0.3209	0.056*	
H5B	0.0867	0.3030	0.2704	0.056*	
C6	0.0963 (3)	0.5244 (9)	0.3406 (4)	0.0441 (15)	
H6A	0.0702	0.6049	0.3045	0.053*	
H6B	0.0867	0.5535	0.3926	0.053*	

### Atomic displacement parameters (Å^2^)

**Table:**

	*U*^11^	*U*^22^	*U*^33^	*U*^12^	*U*^13^	*U*^23^
Zn1	0.0336 (4)	0.0388 (4)	0.0292 (4)	−0.0052 (3)	0.0042 (3)	0.0017 (3)
I1	0.0509 (3)	0.0455 (3)	0.0502 (3)	−0.00351 (19)	0.0210 (2)	−0.00607 (18)
I2	0.0723 (4)	0.0381 (3)	0.0476 (3)	−0.01108 (19)	0.0055 (2)	−0.00284 (17)
N1	0.042 (3)	0.040 (3)	0.031 (2)	−0.002 (2)	0.011 (2)	0.002 (2)
N2	0.057 (4)	0.071 (4)	0.047 (3)	0.020 (3)	0.020 (3)	0.015 (3)
N3	0.033 (2)	0.040 (3)	0.028 (2)	−0.005 (2)	0.0016 (19)	0.004 (2)
N4	0.035 (3)	0.047 (3)	0.030 (2)	−0.004 (2)	0.004 (2)	0.004 (2)
N5	0.042 (3)	0.102 (5)	0.029 (3)	−0.005 (3)	0.002 (2)	0.010 (3)
N6	0.031 (2)	0.046 (3)	0.032 (2)	−0.002 (2)	0.002 (2)	0.008 (2)
C1	0.037 (3)	0.038 (3)	0.035 (3)	−0.004 (3)	0.010 (3)	0.001 (3)
C2	0.066 (4)	0.067 (5)	0.034 (3)	0.008 (4)	0.019 (3)	0.010 (3)
C3	0.041 (3)	0.044 (3)	0.032 (3)	−0.004 (3)	0.007 (3)	0.001 (3)
C4	0.050 (4)	0.089 (6)	0.031 (3)	−0.007 (4)	−0.001 (3)	0.017 (3)
C5	0.037 (3)	0.068 (4)	0.032 (3)	−0.012 (3)	−0.007 (3)	0.009 (3)
C6	0.034 (3)	0.058 (4)	0.040 (3)	0.007 (3)	0.003 (3)	0.016 (3)

### Geometric parameters (Å, °)

**Table:**

Zn1—N1	2.017 (5)	N5—C4	1.285 (9)
Zn1—N4^i^	2.019 (5)	N6—C3	1.333 (7)
Zn1—I1	2.5479 (8)	N6—C4	1.351 (8)
Zn1—I2	2.5523 (9)	N6—C6	1.463 (7)
N1—C1	1.296 (7)	C1—H1A	0.9300
N1—N2	1.369 (7)	C2—H2A	0.9300
N2—C2	1.301 (8)	C3—H3A	0.9300
N3—C2	1.342 (8)	C4—H4A	0.9300
N3—C1	1.350 (7)	C5—C6	1.502 (9)
N3—C5	1.455 (8)	C5—H5A	0.9700
N4—C3	1.301 (7)	C5—H5B	0.9700
N4—N5	1.384 (7)	C6—H6A	0.9700
N4—Zn1^i^	2.019 (5)	C6—H6B	0.9700
			
N1—Zn1—N4^i^	103.68 (19)	N3—C1—H1A	125.2
N1—Zn1—I1	108.78 (14)	N2—C2—N3	111.8 (5)
N4^i^—Zn1—I1	112.08 (14)	N2—C2—H2A	124.1
N1—Zn1—I2	113.03 (14)	N3—C2—H2A	124.1
N4^i^—Zn1—I2	107.90 (14)	N4—C3—N6	110.5 (5)
I1—Zn1—I2	111.19 (3)	N4—C3—H3A	124.7
C1—N1—N2	108.7 (5)	N6—C3—H3A	124.7
C1—N1—Zn1	129.1 (4)	N5—C4—N6	112.3 (6)
N2—N1—Zn1	122.1 (4)	N5—C4—H4A	123.9
C2—N2—N1	105.3 (5)	N6—C4—H4A	123.9
C2—N3—C1	104.5 (5)	N3—C5—C6	113.5 (5)
C2—N3—C5	128.9 (5)	N3—C5—H5A	108.9
C1—N3—C5	126.6 (5)	C6—C5—H5A	108.9
C3—N4—N5	107.7 (5)	N3—C5—H5B	108.9
C3—N4—Zn1^i^	133.9 (4)	C6—C5—H5B	108.9
N5—N4—Zn1^i^	118.4 (4)	H5A—C5—H5B	107.7
C4—N5—N4	105.3 (5)	N6—C6—C5	112.1 (5)
C3—N6—C4	104.2 (5)	N6—C6—H6A	109.2
C3—N6—C6	128.2 (5)	C5—C6—H6A	109.2
C4—N6—C6	127.5 (5)	N6—C6—H6B	109.2
N1—C1—N3	109.6 (5)	C5—C6—H6B	109.2
N1—C1—H1A	125.2	H6A—C6—H6B	107.9
			
N4^i^—Zn1—N1—C1	−131.9 (5)	C1—N3—C2—N2	0.9 (8)
I1—Zn1—N1—C1	−12.5 (5)	C5—N3—C2—N2	−178.9 (6)
I2—Zn1—N1—C1	111.5 (5)	N5—N4—C3—N6	1.5 (7)
N4^i^—Zn1—N1—N2	51.5 (5)	Zn1^i^—N4—C3—N6	177.6 (4)
I1—Zn1—N1—N2	171.0 (4)	C4—N6—C3—N4	−0.9 (7)
I2—Zn1—N1—N2	−65.0 (5)	C6—N6—C3—N4	176.2 (6)
C1—N1—N2—C2	1.0 (8)	N4—N5—C4—N6	0.9 (8)
Zn1—N1—N2—C2	178.2 (5)	C3—N6—C4—N5	−0.1 (8)
C3—N4—N5—C4	−1.4 (7)	C6—N6—C4—N5	−177.1 (6)
Zn1^i^—N4—N5—C4	−178.3 (5)	C2—N3—C5—C6	−101.4 (8)
N2—N1—C1—N3	−0.4 (7)	C1—N3—C5—C6	78.8 (7)
Zn1—N1—C1—N3	−177.4 (4)	C3—N6—C6—C5	−93.3 (7)
C2—N3—C1—N1	−0.3 (7)	C4—N6—C6—C5	83.1 (8)
C5—N3—C1—N1	179.6 (5)	N3—C5—C6—N6	63.8 (7)
N1—N2—C2—N3	−1.2 (9)		

Symmetry codes: (i) −*x*+1/2, −*y*+1/2, −*z*+1.

## References

[bb1] Haasnoot, J. G. (2000). *Coord. Chem. Rev.* **200–202**, 131–185.

[bb2] Habit, H. A., Hoffmann, A., Hoppe, H. A., Steinfeld, G. & Janiak, C. (2009). *Inorg. Chem.* **48**, 2166–2180. 10.1021/ic802069k 19235976

[bb3] Jacobson, R. (1998). *REQAB* Private communication to the Rigaku Corporation, Tokyo, Japan.

[bb4] Li, B.-L., Li, B.-Z., Zhu, X., Zhu, L.-M. & Zhang, Y. (2003). *Acta Cryst.* C**59**, m350–m351. 10.1107/s0108270103012435 12944641

[bb5] Rigaku (2000). *CrystalClear.* Rigaku Corporation, Tokyo, Japan.

[bb6] Sheldrick, G. M. (2008). *Acta Cryst.* A**64**, 112–122. 10.1107/S0108767307043930 18156677

[bb7] Zhang, Y.-M., Zhang, Y.-P., Li, B.-L. & Zhang, Y. (2007). *Acta Cryst.* C**63**, m120–m122. 10.1107/S0108270107005215 17339706

[bb8] Zhu, X., Li, B.-Z., Zhou, J.-H., Li, B.-L. & Zhang, Y. (2004). *Acta Cryst.* C**60**, m191–m193. 10.1107/S0108270104005542 15071215

